# 1,10-Bis[2-(prop-1-en­yl)phen­oxy]deca­ne

**DOI:** 10.1107/S1600536811055061

**Published:** 2012-01-07

**Authors:** Abel M. Maharramov, Musa R. Bayramov, Gunay M. Mehdiyeva, Shahnaz B. Hoseinzadeh, Bahruz A. Rashidov

**Affiliations:** a Baku State University, Z. Khalilov St. 23, Baku AZ-1148, Azerbaijan

## Abstract

The complete molecule of the title compound, C_28_H_38_O_2_, is generated by a crystallographic centre of symmetry. The molecular conformation displays an intra­molecular C—H⋯π inter­action.

## Related literature

For general background to the synthesis, see: Wadher *et al.* (2009) ▶. For the use of cross-linked polymers in the synthesis of multifunctional monomers, see: Starvin & Rao (2004) ▶. For their applications as polymeric sorbents and in the preparation of laser composites, see: Kazuya *et al.* (2000 ▶); Ryusuke & Kazufumi (2001) ▶. For a related structure, see: Bayramov *et al.* (2011 ▶).
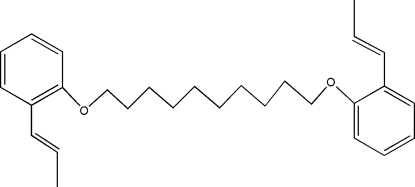


## Experimental

### 

#### Crystal data

C_28_H_38_O_2_
*M* *_r_* = 406.58
Monoclinic, 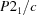

*a* = 5.4084 (6) Å
*b* = 12.2076 (14) Å
*c* = 19.391 (2) Å
β = 92.025 (2)°
*V* = 1279.5 (3) Å^3^
*Z* = 2
Mo *K*α radiation
μ = 0.06 mm^−1^
*T* = 296 K
0.30 × 0.20 × 0.20 mm

#### Data collection

Bruker APEXII CCD diffractometer
Absorption correction: multi-scan (*SADABS*; Sheldrick, 1998 ▶) *T*_min_ = 0.981, *T*_max_ = 0.987
13946 measured reflections
3057 independent reflections
1914 reflections with *I* > 2σ(*I*)
*R*_int_ = 0.025

#### Refinement

*R*[*F* ^2^ > 2σ(*F* ^2^)] = 0.069
*wR*(*F* ^2^) = 0.245
*S* = 1.00
3057 reflections
136 parameters
H-atom parameters constrained
Δρ_max_ = 0.60 e Å^−3^
Δρ_min_ = −0.26 e Å^−3^

### 

Data collection: *APEX2* (Bruker, 2005 ▶); cell refinement: *SAINT-Plus* (Bruker, 2001 ▶); data reduction: *SAINT-Plus*; program(s) used to solve structure: *SHELXTL* (Sheldrick, 2008) ▶; program(s) used to refine structure: *SHELXTL* ▶; molecular graphics: *SHELXTL*; software used to prepare material for publication: *SHELXTL*.

## Supplementary Material

Crystal structure: contains datablock(s) global, I. DOI: 10.1107/S1600536811055061/kp2373sup1.cif

Structure factors: contains datablock(s) I. DOI: 10.1107/S1600536811055061/kp2373Isup2.hkl

Supplementary material file. DOI: 10.1107/S1600536811055061/kp2373Isup3.cml

Additional supplementary materials:  crystallographic information; 3D view; checkCIF report

## Figures and Tables

**Table 1:** Hydrogen-bond geometry (Å, °) *Cg*1 is the centroid of the C1–C6 ring.

*D*—H⋯*A*	*D*—H	H⋯*A*	*D*⋯*A*	*D*—H⋯*A*
C7—H7*B*⋯*Cg*1	0.97	2.65	2.396 (3)	143

**Fig. 1.**
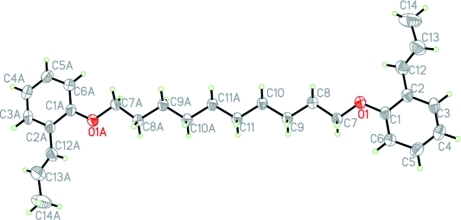
The molecular structure of the title compound with the atomic numbering scheme. Displacement ellipsoids were drawn at the 30% probability level.

**Fig. 2.**
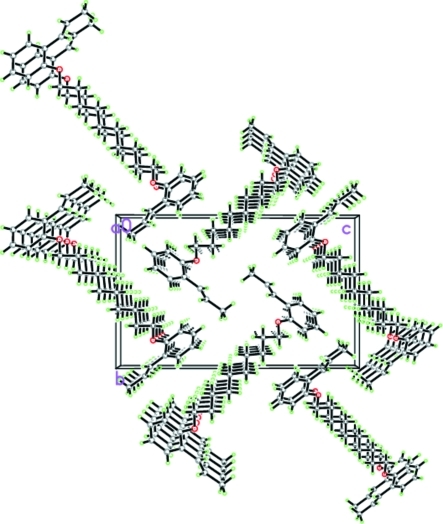
Packing of chains in the unit cell.

## supplementary crystallographic information

### Comment

Operational cross-linked polymers have been used for synthesis of multifunctional monomers (Starvin *et al.* (2004). These polymers are useful in many applications such as polymeric sorbents and preparing the laser composites (Kazuya *et al.*, 2000); Ryusuke & Kazufumi (2001). In practice, for obtaining polymers of improved functional properties, polymerical transformations are carried out. However, preparation of such cross-linked copolymers have some difficulties related to monomers high reactivity (for example, divinybenzene) and other physico-chemical properties. Therefore, synthesis of multifunctional monomers based on the alkenylphenols is rather important. The authors were synthesised the multifunctional monomers (Bayramov *et al.*, 2011), that can be used in preparation of cross-linked copolymers as a sorbent for heavy metals.

The molecule of title compound, C_28_H_38_O_2_, (I), reveals a crystallographic inversion centre at the mid-point of the central C—C bond (Fig. 1). An asymmetric unit comprises a half of the molecule. The crystal packing displays intramolecular C—H···O hydrogen bonds and C—H···π interaction (Fig. 2, Table 1). The molecule has long chain of (CH_2_) groups, and so, the polymers based on this monomer are capable to adsorbed heavy metal ions.

### Experimental

2-Propenylphenol (0.015 mol, 2 g) and KOH (0.015 mol, 0.84 g) were dissolved in 6 mL of 2-propanol, then 1,10-dibromedecane (0.006 mol, 1.8 g) was added to this solution. This mixture was stirred at 353 K for 30 m. The desired compounds with yield 2.43 g (99.1%) was filtered and washed with acetone and recrystallised to obtain colourless crystals. T_mp_ = 326 K. The structure of the reported compound - 1,10-bis{2(1-propenyl)phenoxy}decane, was also proved by NMR-spectroscopy. FT-NMR (acetone-d^6^, p.p.m.), ^1^H: 1.92 d (6*H*,CH_3_); 2.05 t (4*H*, CH_2_); 4.16 t (4*H*, OCH_2_); 6.13 m (2*H*, CH=); 6.67–7.2 m (8*H*, 2Ar); 7.3 d (2*H*,CH=). ^13^C: 18.5; 26.1; 67.1; 112.3; 121.4; 124.4; 126.0; 127.1; 127.3; 127.5; 156.0.

### Refinement

The hydrogen atoms were placed at calculated positions and refined in the riding mode with fixed isotropic displacement parameters [*U*_iso_(H) = 1.2Ueq(C)].

### Crystal data

**Table:**

C_28_H_38_O_2_	*F*(000) = 444
*M**_r_* = 406.58	*D*_x_ = 1.055 Mg m^−^^3^
Monoclinic, *P*2_1_/*c*	Mo *K*α radiation, λ = 0.71073 Å
Hall symbol: -P 2ybc	Cell parameters from 3093 reflections
*a* = 5.4084 (6) Å	θ = 2.7–25.5°
*b* = 12.2076 (14) Å	µ = 0.06 mm^−^^1^
*c* = 19.391 (2) Å	*T* = 296 K
β = 92.025 (2)°	Prism, colourless
*V* = 1279.5 (3) Å^3^	0.30 × 0.20 × 0.20 mm
*Z* = 2	

### Data collection

**Table:**

Bruker APEXII CCD diffractometer	3057 independent reflections
Radiation source: fine-focus sealed tube	1914 reflections with *I* > 2σ(*I*)
graphite	*R*_int_ = 0.025
phi and ω scans	θ_max_ = 28.0°, θ_min_ = 2.0°
Absorption correction: multi-scan (*SADABS*; Sheldrick, 1998)	*h* = −7→7
*T*_min_ = 0.981, *T*_max_ = 0.987	*k* = −16→16
13946 measured reflections	*l* = −25→25

### Refinement

**Table:**

Refinement on *F*^2^	Primary atom site location: structure-invariant direct methods
Least-squares matrix: full	Secondary atom site location: difference Fourier map
*R*[*F*^2^ > 2σ(*F*^2^)] = 0.069	Hydrogen site location: difference Fourier map
*wR*(*F*^2^) = 0.245	H-atom parameters constrained
*S* = 1.00	*w* = 1/[σ^2^(*F*_o_^2^) + (0.1494*P*)^2^ + 0.1404*P*] where *P* = (*F*_o_^2^ + 2*F*_c_^2^)/3
3057 reflections	(Δ/σ)_max_ < 0.001
136 parameters	Δρ_max_ = 0.60 e Å^−^^3^
0 restraints	Δρ_min_ = −0.26 e Å^−^^3^

### Special details

**Table:**

Geometry. All e.s.d.'s (except the e.s.d. in the dihedral angle between two l.s. planes) are estimated using the full covariance matrix. The cell e.s.d.'s are taken into account individually in the estimation of e.s.d.'s in distances, angles and torsion angles; correlations between e.s.d.'s in cell parameters are only used when they are defined by crystal symmetry. An approximate (isotropic) treatment of cell e.s.d.'s is used for estimating e.s.d.'s involving l.s. planes.
Refinement. Refinement of *F*^2^ against ALL reflections. The weighted *R*-factor *wR* and goodness of fit *S* are based on *F*^2^, conventional *R*-factors *R* are based on *F*, with *F* set to zero for negative *F*^2^. The threshold expression of *F*^2^ > σ(*F*^2^) is used only for calculating *R*-factors(gt) *etc*. and is not relevant to the choice of reflections for refinement. *R*-factors based on *F*^2^ are statistically about twice as large as those based on *F*, and *R*- factors based on ALL data will be even larger.

### Fractional atomic coordinates and isotropic or equivalent isotropic displacement parameters (Å^2^)

**Table:**

	*x*	*y*	*z*	*U*_iso_*/*U*_eq_	
O1	0.3626 (3)	0.30774 (11)	0.33897 (7)	0.0679 (4)	
C1	0.5218 (3)	0.32178 (16)	0.28696 (10)	0.0594 (5)	
C2	0.6858 (4)	0.41008 (15)	0.29461 (11)	0.0623 (5)	
C3	0.8580 (4)	0.42419 (19)	0.24299 (13)	0.0759 (6)	
H3A	0.9706	0.4816	0.2470	0.091*	
C4	0.8663 (5)	0.3562 (2)	0.18664 (13)	0.0807 (7)	
H4A	0.9832	0.3675	0.1533	0.097*	
C5	0.7018 (5)	0.2721 (2)	0.18001 (12)	0.0801 (7)	
H5A	0.7052	0.2265	0.1417	0.096*	
C6	0.5292 (4)	0.25403 (19)	0.23007 (10)	0.0709 (6)	
H6A	0.4182	0.1961	0.2253	0.085*	
C7	0.2015 (3)	0.21479 (15)	0.33632 (10)	0.0583 (5)	
H7A	0.2977	0.1480	0.3336	0.070*	
H7B	0.0917	0.2190	0.2958	0.070*	
C8	0.0534 (3)	0.21399 (15)	0.40044 (10)	0.0569 (5)	
H8A	−0.0470	0.2797	0.4018	0.068*	
H8B	0.1649	0.2142	0.4407	0.068*	
C9	−0.1122 (3)	0.11440 (15)	0.40254 (9)	0.0573 (5)	
H9A	−0.0101	0.0492	0.4008	0.069*	
H9B	−0.2211	0.1145	0.3617	0.069*	
C10	−0.2681 (3)	0.10799 (15)	0.46559 (10)	0.0572 (5)	
H10A	−0.3772	0.1710	0.4661	0.069*	
H10B	−0.1602	0.1116	0.5066	0.069*	
C11	−0.4231 (3)	0.00446 (16)	0.46864 (10)	0.0590 (5)	
H11A	−0.5320	0.0015	0.4279	0.071*	
H11B	−0.3137	−0.0584	0.4673	0.071*	
C12	0.6767 (5)	0.48117 (17)	0.35572 (13)	0.0801 (7)	
H12A	0.5316	0.4780	0.3800	0.096*	
C13	0.8412 (7)	0.5460 (2)	0.37940 (17)	0.1110 (10)	
H13A	0.9898	0.5505	0.3569	0.133*	
C14	0.8081 (10)	0.6169 (3)	0.4428 (2)	0.1568 (18)	
H14A	0.9548	0.6596	0.4516	0.235*	
H14B	0.7791	0.5710	0.4819	0.235*	
H14C	0.6693	0.6649	0.4350	0.235*	

### Atomic displacement parameters (Å^2^)

**Table:**

	*U*^11^	*U*^22^	*U*^33^	*U*^12^	*U*^13^	*U*^23^
O1	0.0720 (9)	0.0654 (8)	0.0673 (9)	−0.0168 (7)	0.0141 (7)	−0.0044 (6)
C1	0.0610 (11)	0.0573 (10)	0.0602 (10)	−0.0039 (8)	0.0041 (8)	0.0110 (8)
C2	0.0671 (11)	0.0512 (10)	0.0685 (11)	−0.0033 (8)	0.0004 (9)	0.0138 (8)
C3	0.0764 (13)	0.0648 (12)	0.0871 (15)	−0.0092 (10)	0.0102 (11)	0.0256 (11)
C4	0.0881 (16)	0.0832 (15)	0.0720 (14)	0.0012 (12)	0.0210 (12)	0.0203 (11)
C5	0.0949 (17)	0.0840 (15)	0.0622 (12)	−0.0005 (13)	0.0131 (11)	0.0034 (10)
C6	0.0767 (13)	0.0731 (13)	0.0631 (12)	−0.0117 (10)	0.0051 (10)	0.0010 (9)
C7	0.0580 (10)	0.0544 (10)	0.0625 (10)	−0.0083 (8)	0.0034 (8)	0.0039 (8)
C8	0.0544 (10)	0.0550 (10)	0.0613 (10)	−0.0001 (8)	0.0049 (8)	0.0041 (8)
C9	0.0514 (10)	0.0606 (10)	0.0599 (10)	−0.0024 (8)	0.0044 (8)	0.0050 (8)
C10	0.0482 (9)	0.0611 (10)	0.0626 (10)	−0.0007 (8)	0.0062 (8)	0.0055 (8)
C11	0.0486 (10)	0.0647 (11)	0.0640 (11)	−0.0019 (8)	0.0066 (8)	0.0067 (8)
C12	0.0940 (17)	0.0545 (11)	0.0915 (16)	−0.0099 (11)	0.0002 (13)	0.0077 (10)
C13	0.122 (2)	0.0941 (19)	0.116 (2)	−0.0182 (18)	−0.0054 (19)	−0.0093 (16)
C14	0.229 (5)	0.101 (2)	0.137 (3)	−0.006 (3)	−0.048 (3)	−0.037 (2)

### Geometric parameters (Å, °)

**Table:**

O1—C1	1.360 (2)	C8—H8B	0.9700
O1—C7	1.430 (2)	C9—C10	1.511 (2)
C1—C6	1.380 (3)	C9—H9A	0.9700
C1—C2	1.401 (3)	C9—H9B	0.9700
C2—C3	1.402 (3)	C10—C11	1.519 (3)
C2—C12	1.471 (3)	C10—H10A	0.9700
C3—C4	1.374 (4)	C10—H10B	0.9700
C3—H3A	0.9300	C11—C11^i^	1.502 (4)
C4—C5	1.361 (4)	C11—H11A	0.9700
C4—H4A	0.9300	C11—H11B	0.9700
C5—C6	1.388 (3)	C12—C13	1.265 (4)
C5—H5A	0.9300	C12—H12A	0.9300
C6—H6A	0.9300	C13—C14	1.519 (5)
C7—C8	1.503 (3)	C13—H13A	0.9300
C7—H7A	0.9700	C14—H14A	0.9600
C7—H7B	0.9700	C14—H14B	0.9600
C8—C9	1.511 (3)	C14—H14C	0.9600
C8—H8A	0.9700		
			
C1—O1—C7	118.31 (15)	C8—C9—C10	114.29 (16)
O1—C1—C6	123.66 (17)	C8—C9—H9A	108.7
O1—C1—C2	115.70 (17)	C10—C9—H9A	108.7
C6—C1—C2	120.63 (18)	C8—C9—H9B	108.7
C1—C2—C3	116.96 (19)	C10—C9—H9B	108.7
C1—C2—C12	120.00 (19)	H9A—C9—H9B	107.6
C3—C2—C12	123.02 (19)	C9—C10—C11	113.51 (16)
C4—C3—C2	122.3 (2)	C9—C10—H10A	108.9
C4—C3—H3A	118.8	C11—C10—H10A	108.9
C2—C3—H3A	118.8	C9—C10—H10B	108.9
C5—C4—C3	119.4 (2)	C11—C10—H10B	108.9
C5—C4—H4A	120.3	H10A—C10—H10B	107.7
C3—C4—H4A	120.3	C11^i^—C11—C10	114.5 (2)
C4—C5—C6	120.5 (2)	C11^i^—C11—H11A	108.6
C4—C5—H5A	119.8	C10—C11—H11A	108.6
C6—C5—H5A	119.8	C11^i^—C11—H11B	108.6
C1—C6—C5	120.2 (2)	C10—C11—H11B	108.6
C1—C6—H6A	119.9	H11A—C11—H11B	107.6
C5—C6—H6A	119.9	C13—C12—C2	128.2 (3)
O1—C7—C8	108.50 (15)	C13—C12—H12A	115.9
O1—C7—H7A	110.0	C2—C12—H12A	115.9
C8—C7—H7A	110.0	C12—C13—C14	123.3 (4)
O1—C7—H7B	110.0	C12—C13—H13A	118.4
C8—C7—H7B	110.0	C14—C13—H13A	118.4
H7A—C7—H7B	108.4	C13—C14—H14A	109.5
C7—C8—C9	111.18 (16)	C13—C14—H14B	109.5
C7—C8—H8A	109.4	H14A—C14—H14B	109.5
C9—C8—H8A	109.4	C13—C14—H14C	109.5
C7—C8—H8B	109.4	H14A—C14—H14C	109.5
C9—C8—H8B	109.4	H14B—C14—H14C	109.5
H8A—C8—H8B	108.0		
			
C7—O1—C1—C6	3.0 (3)	C2—C1—C6—C5	0.7 (3)
C7—O1—C1—C2	−176.00 (16)	C4—C5—C6—C1	0.4 (4)
O1—C1—C2—C3	177.78 (17)	C1—O1—C7—C8	177.33 (15)
C6—C1—C2—C3	−1.3 (3)	O1—C7—C8—C9	−177.21 (15)
O1—C1—C2—C12	−0.6 (3)	C7—C8—C9—C10	−179.81 (15)
C6—C1—C2—C12	−179.6 (2)	C8—C9—C10—C11	−176.97 (15)
C1—C2—C3—C4	0.8 (3)	C9—C10—C11—C11^i^	179.19 (18)
C12—C2—C3—C4	179.1 (2)	C1—C2—C12—C13	161.6 (3)
C2—C3—C4—C5	0.3 (4)	C3—C2—C12—C13	−16.7 (4)
C3—C4—C5—C6	−0.9 (4)	C2—C12—C13—C14	179.0 (3)
O1—C1—C6—C5	−178.27 (19)		

Symmetry codes: (i) −*x*−1, −*y*, −*z*+1.

### Hydrogen-bond geometry (Å, °)

**Table:**

Cg1 is the centroid of the C1–C6 ring.

**Table:**

*D*—H···*A*	*D*—H	H···*A*	*D*···*A*	*D*—H···*A*
C12—H12···O1	0.93 (3)	2.40 (3)	2.727 (3)	101 (3)
C7—H7B···Cg1	0.97	2.65	2.396 (3)	143

## References

[bb1] Bayramov, M. R., Maharramov, A. M., Mehdiyeva, G. M., Hoseinzadeh, S. B. & Askerov, R. K. (2011). *Acta Cryst.* E**67**, o1478. 10.1107/S1600536811018538 PMC3120590 21754847

[bb2] Bruker (2001). *SAINT-Plus* Bruker AXS Inc., Madison, Wisconsin, USA.

[bb3] Bruker (2005). *APEX2* Bruker AXS Inc., Madison, Wisconsin, USA.

[bb4] Kazuya, U., Yutaka, A. & Koji, S. (2000). Jpn Patent No. 1117002.

[bb5] Ryusuke, U. & Kazufumi, S. (2001). US Patent No 6284430.

[bb6] Sheldrick, G. M. (1998). *SADABS* Bruker AXS Inc., Madison, Wisconsin, USA.

[bb7] Sheldrick, G. M. (2008). *Acta Cryst.* A**64**, 112–122. 10.1107/S0108767307043930 18156677

[bb9] Starvin, A. M. & Rao, T. P. (2004). *Talanta*, **63**, 225–232. 10.1016/j.talanta.2003.11.001 18969421

[bb8] Wadher, S. J., Puranik, M. P., Karande, N. A. & Yeole, P. G. (2009). *Int. J. Pharm. Tech. Res.* **1**, 22–33.

